# Short-term outcome of Polytetrafluoroethylene Membrane Valve *versus* Transannular Pericardial patch Reconstruction of Right Ventricular Outflow Tract in Tetralogy of Fallot: a Randomized Controlled Trial

**DOI:** 10.21470/1678-9741-2020-0059

**Published:** 2021

**Authors:** Sanjib Rawat, Vivek Jaswal, Shyam Kumar Singh Thingnam, Harkant Singh, Sachin Mahajan, Reuben Lamiaki Kynta, Goverdhan Dutt Puri, Manoj Kumar Rohit

**Affiliations:** 1 Department of Cardiovascular and Thoracic Surgery, Postgraduate Institute of Medical Education and Research (PGIMER), Chandigarh, India.; 2 Department of Anaesthesia and Critical Care, Postgraduate Institute of Medical Education and Research (PGIMER), Chandigarh, India.; 3 Department of Cardiology, Postgraduate Institute of Medical Education and Research (PGIMER), Chandigarh, India.

**Keywords:** Tetralogy of Fallot, Polytetrafluoroethylene, Bicuspid, Echocardiography, Pulmonary valve insufficiency, Airway extubation, Central venous pressure

## Abstract

**Introduction:**

Reconstruction of right ventricular outflow tract during primary repair of tetralogy of Fallot often requires the placement of a transannular patch which results in pulmonary regurgitation (PR). We compared the short-term outcomes of bicuspid polytetrafluoroethylene membrane valve *versus* transannular pericardial patch reconstruction of the right ventricular outflow tract.

**Methods:**

Thirty consecutive patients undergoing primary repair of tetralogy of Fallot were randomly allocated to two groups - polytetrafluoroethylene valve (PTFEV) group (n=15) and transannular pericardial patch (TAP) group (n=15). The two groups had similar preoperative demographic characteristics. We compared the short-term clinical and echocardiographic outcomes between these groups. The transthoracic echocardiographic follow-up was performed at one week, one month and six months after surgery.

**Results:**

The PTFEV group had significantly lower central venous pressure in the immediate postoperative period compared to the TAP group (7.60±2.06 *vs*. 10.13±1.73, *P*=0.002). Extubation time was significantly shorter in the PTFEV group compared to the TAP group (12.93±7.55 hrs *vs*. 22.23±15.11 hrs, *P*=0.04). PR in the PTFEV group was absent in five patients at 24 hours post-surgery. At the study endpoint, PR was absent in six, trivial in one and mild in eight patients in the PTFEV group compared to TAP group, where all 15 patients had severe PR.

**Conclusion:**

The bicuspid polytetrafluoroethylene membrane valves significantly decrease the central venous pressure in the immediate postoperative period, facilitate early extubation and, thus, prevent ventilator-related comorbidities. They achieve a high degree of pulmonary competence and do not increase the right ventricular outflow tract gradient in short-term follow-up.

**Table:** 

Abbreviations, acronyms & symbols				
BT	= Blalock-Taussig		RV	= Right ventricle
CPB	= Cardiopulmonary bypass		RVOT	= Right ventricular outflow tract
CVP	= Central venous pressure		SPSS	= Statistical Package for the Social Sciences
ICU	= Intensive care unit		SVC	= Superior vena cava
LV	= Left ventricle		TAP	= Transannular pericardial patch
MAPCAs	= Major aortopulmonary collateral arteries		TAPSE	= Tricuspid annular plane systolic excursion
MPA	= Main pulmonary artery		TOF	= Tetralogy of Fallot
PR	= Pulmonary regurgitation		VIS	= Vasoactive inotropic score
PTFEV	= Polytetrafluoroethylene valve		VSD	= Ventricular septal defect

## INTRODUCTION

Pulmonary regurgitation (PR) is a common sequel of transannular pericardial patch (TAP) augmentation of right ventricular outflow tract (RVOT) in patients with tetralogy of Fallot (TOF) after intracardiac repair. The free PR is responsible for late postoperative right ventricle (RV) dysfunction, arrhythmia and reduced functional capacity. Several strategies have been tried to minimize the PR with the intraoperative use of valved conduits, pericardial valves, polytetrafluoroethylene (PTFE) monocuspid valve, and PTFE bicuspid pulmonary valve^[1-3]^. The use of valved conduits has the disadvantage of lack of durability, lack of growth and valvular dysfunction. The use of pericardial valve has failed to prevent the development of PR. Use of PTFE monocuspid valve has shown mixed results, most of which are unfavorable^[2]^. PTFE bicuspid pulmonary valve has shown some promising results^[3]^. Few observational series have shown a significant decrease in PR over short-to-medium term when PTFE bicuspid pulmonary valve was incorporated during repair of TOF, resulting in better outcome^[3]^. In this study, we compared the early clinical and echocardiographic outcomes among the patients undergoing primary repair of TOF using TAP with bicuspid PTFE membrane valve versus TAP alone, with an emphasis on early postoperative course and the development of PR over short-term period.

## METHODS

After approval by the institutional ethics committee and written informed consent from the patients, 30 consecutive patients undergoing primary intracardiac repair of TOF with reconstruction of RVOT were enrolled in this study from July 1st 2016 to December 31st 2017. One patient had previously undergone palliative treatment with a PTFE systemic-pulmonary shunt via left thoracotomy. All patients were operated on by an experienced cardiac surgeon. Patients were randomly allocated to two groups as per computer-generated randomization sequence, once the decision to perform a TAP repair had been confirmed in the operating room - PTFE valve (PTFEV) group, which included patients who had 0.1 mm PTFE membrane bicuspid valve insertion along with transannular patch augmentation of RVOT and TAP group, which included patients who had transannular patch augmentation of RVOT alone. Patients with confluent, good-sized branch pulmonary arteries undergoing elective primary repair of TOF requiring transannular patch with or without previous shunt(s) were included in the study. Exclusion criteria included neonates and infants, absent pulmonary valve, pulmonary atresia, branch pulmonary artery stenosis, patients requiring conduits to bridge pulmonary artery deficiency, residual ventricular septal defect and emergency surgery (<24 hours after admission) in view of sudden cardiac decompensation/desaturation.

Surgical technique remained constant. Autologous pericardium harvesting and treatment with 0.6% glutaraldehyde for six minutes, adequate dissection of aorta and branch pulmonary arteries, dissection of any previous shunt(s), major aortopulmonary collateral arteries (MAPCAs) dissection/clipping between aorta and superior vena cava (SVC) and along mediastinal pleura, bicaval venous cannulation, full cardiopulmonary bypass (CPB) support, clipping of any patent ductus arteriosus/previous shunt(s), left ventricle (LV) venting through the right superior pulmonary vein or patent foramen ovale, moderate hypothermia, cold blood Del Nido cardioplegia and closure of the ventricle septal defect before RVOT reconstruction. The ventricular septal defect (VSD) was closed via transatrial approach in all patients. No patient required tricuspid valve septal leaflet takedown for exposure of VSD. Hypertrophied obstructing septal and parietal muscle bands were excised through the right atrium. The RV outlet area was exposed with the aid of retraction sutures. The pulmonary valve was inspected, and the annulus was sized with a Hegar dilator. The small pulmonary annulus was divided, and the incision was extended to the infundibulum, saving the RV conal branch. If the native pulmonary valve was severely dysplastic, it was excised in toto; otherwise, the pliable leaflets were retained (especially the posterior one). Completion coring was done through the infundibulum. Division of fused commissures and release of tethering to main pulmonary artery (MPA) was done. Sizing of the pericardial patch required was fashioned by measuring it over an adequate sized Hegar dilator (according to the nomogram and the Z value) in such a way that it will comprise two thirds of the reconstituted circumference of the pulmonary annulus and MPA with an additional 2 mm margin for suturing on each side of RVOT and main pulmonary artery.

Bicuspid valves were constructed using a 0.1-mm PTFE membrane cut as a sector of a circle with the radius of the circle equaling the length from the distal posterior wall of the pulmonary artery just before bifurcation to the proximal end of the ventriculotomy incision in the RVOT (Figure 1A). The length of the free edge of the wing was measured from the vertex of ventriculotomy incision to pulmonary annulus (Figure 1B). The pericardial patch augmentation of RVOT and MPA was started at the distal end of the incision, extending to the left pulmonary artery (point 1) with 6-0 polypropylene and carried proximally on both sides to points 2 and 3 (Figure 1C). This sector was folded and the mid part of the curved edge was sutured distally to the mid part of posterior wall of MPA just before bifurcation (point 4) with a pledgeted 6-0 polypropylene suture (Figure 1C). Then the proximal end was sutured to the myocardium at the vertex of the ventriculotomy (point 5) with pledgeted 5-0 polypropylene suture (Figure 1C). This allows the patch to be shortened, if it is too large proximally, and then the 2 sides are adjusted for width to give a higher open valve in the patient in whom more augmentation of the outlet is required. The height of the PTFE membrane cusps was measured by pulling the PTFE cusps up to the middle of the pericardial patch. The free-edge angles of the wings were fixed to the RVOT-MPA junction at pulmonary annulus (points 6 and 7) with 5-0 polypropylene suture on both sides (Figure 1C). The pericardial patch suturing was then continued proximally on both sides of the main pulmonary artery till the pulmonary annulus (points 6 and 7) (Figure 1D). Now the pericardial patch (point 8), PTFE membrane (point 5) and infundibulotomy margin (from point 5) were sutured in a single layer in continuous fashion starting from proximal to distal extent (from point 5 to point 6) with pledgeted 5-0 polypropylene suture already placed before at point 5 (Figure 1D). The same technique was repeated on the other side from point 5 to point 7. At the level of pulmonary annulus, the sutures were tied to the distal sutures used to fix the pericardial patch to MPA to complete the repair (Figure 1E). During systole, the valve cusps appose in midline, allowing blood to flow through it, and during diastole, the valve cusps are filled with blood, thereby apposing it to the pulmonary wall and preventing regurgitation (Figure 1F and 1G). Routine weaning from CPB and closure were undertaken. Intensive care unit (ICU) team was blinded to the study, so that there was no bias in the extubation strategy.

**Fig. 1A:**
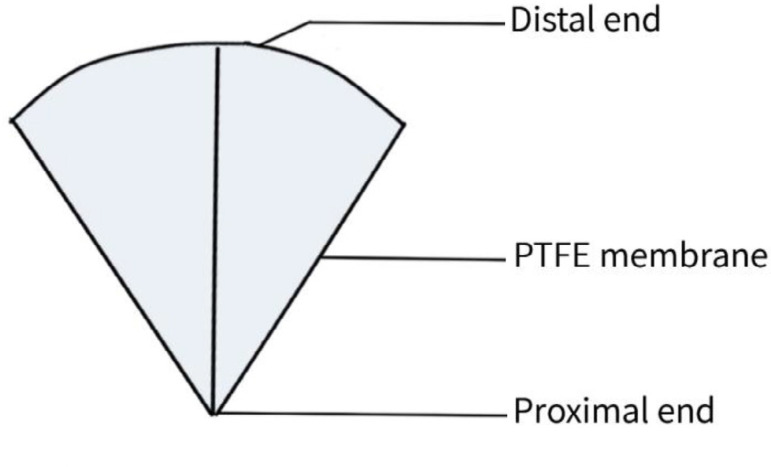
Tailored PTFE membrane. PTFE=polytetrafluoroethylene

**Fig. 1B:**
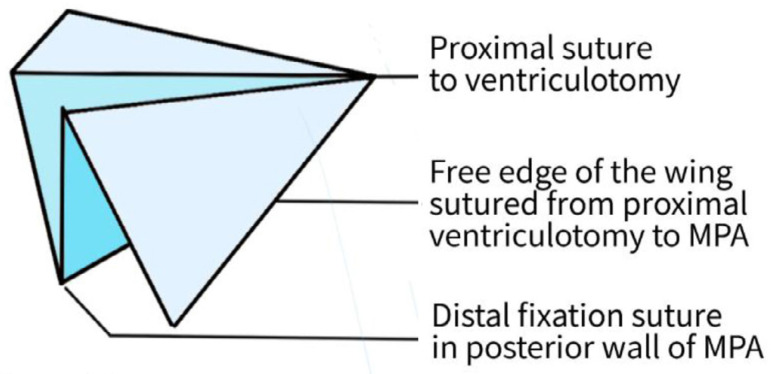
Design of the PTFE membrane valve. MPA=main pulmonary artery

**Fig. 1C:**
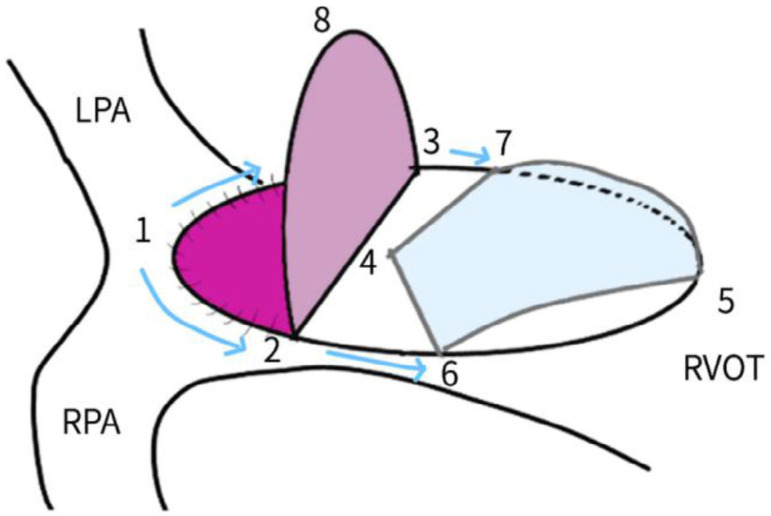
Pericardial patch augmentation started at the distal end of the incision (point 1) and continued proximally on both sides. Distal fixation suture in the middle of the posterior wall of main pulmonary artery (point 4) 5 mm proximal to the right pulmonary artery origin. Proximal suture from the vertex of ventriculotomy to the proximal end of PTFE membrane (point 5). The free-edge angles of the wing fixed to the RVOT-MPA junction at pulmonary annulus (points 6 and 7). LPA=left pulmonary artery; MPA=main pulmonary artery; PTFE=polytetrafluoroethylene; RPA=right pulmonary artery; RVOT=right ventricular outflow tract

**Fig. 1D:**
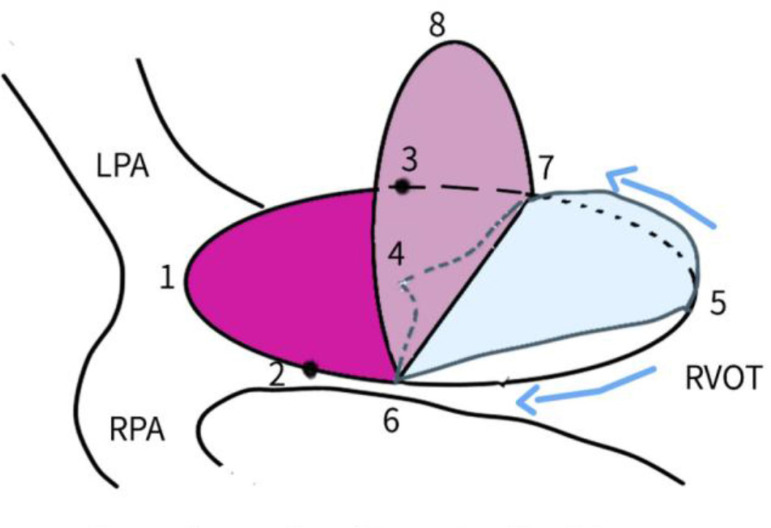
Pericardial patch suturing continued proximally on both sides of the main pulmonary artery till pulmonary annulus (points 6 and 7). Pericardial patch (point 8), PFTE membrane (point 5) and infundibulotomy margin (starting from point 5) were sutured in a single layer in a continuous fashion from proximal to distal extent, starting from point 5 to point 6 and then from point 5 to point 7. LPA=left pulmonary artery; PTFE=polytetrafluoroethylene; RPA=right pulmonary artery; RVOT=right ventricular outflow tract

**Fig. 1E:**
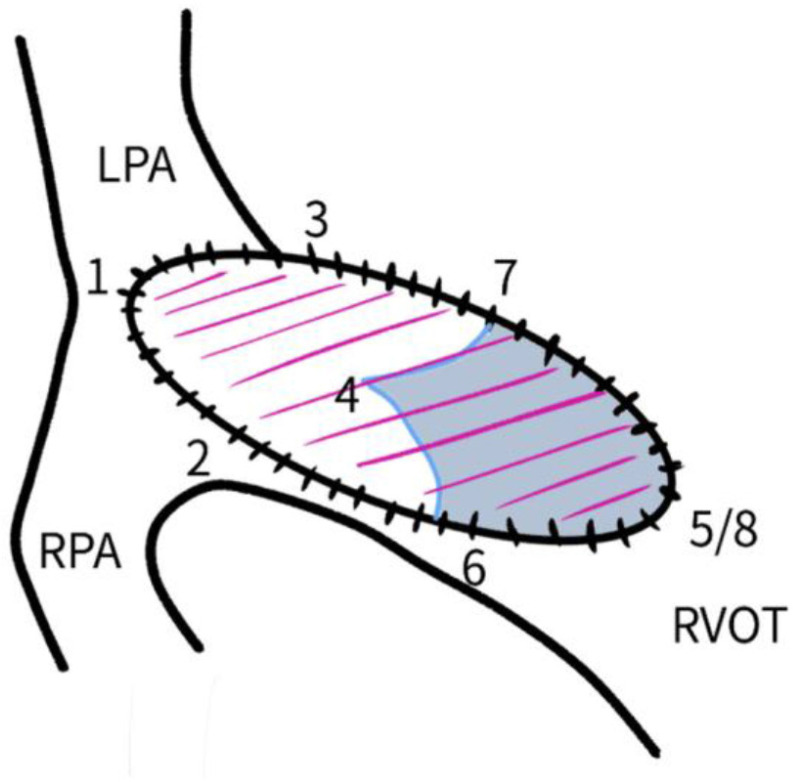
At the level of the pulmonary annulus (points 6 and 7), the sutures were tied to the distal sutures used to fix the pericardial patch to MPA to complete the repair. LPA=left pulmonary artery; MPA=main pulmonary artery; RPA=right pulmonary artery; RVOT=right ventricular outflow tract

**Fig. 1F:**
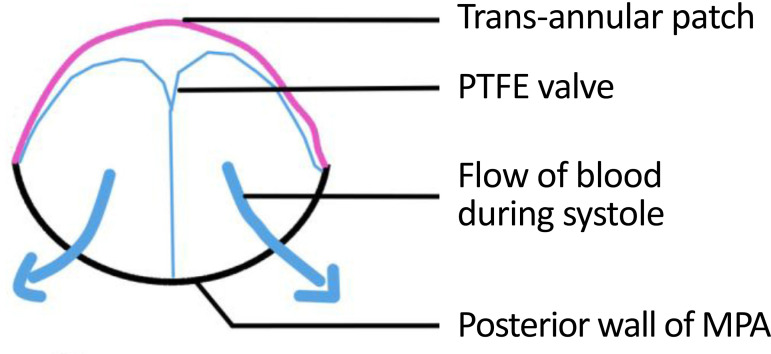
Cross-sectional view of PTFE membrane valve showing blood flow during systole. MPA=main pulmonary artery; PTFE=polytetrafluoroethylene

**Fig. 1G:**
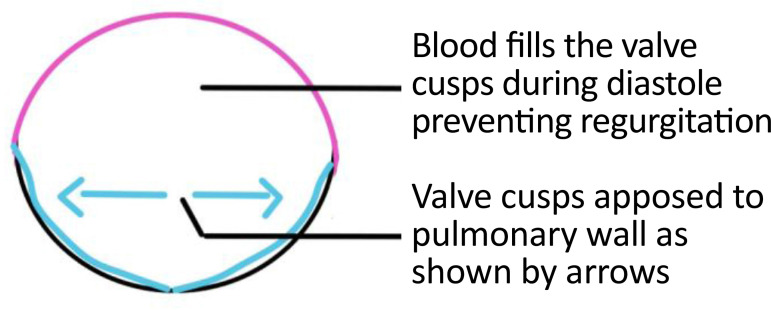
Cross-sectional view of PTFE membrane valve during diastole. PTFE=polytetrafluoroethylene

We compared the short-term clinical and echocardiographic outcomes between these groups. Intraoperative assessment was done by transesophageal echocardiography. Postoperative immediate outcome included central venous pressure in the immediate postoperative period, need for inotropes, duration of mechanical ventilation, length of ICU stay and mortality. The need for inotropes was assessed in terms of the standard inotropic score^[4]^ as follows: $\mathrm{Inotropic}\;\mathrm{score}=\mathrm{Dose}\;\mathrm{in}\;\mathrm{mcg}/\mathrm{kg}/\min\;\mathrm{of}\;\left(\mathrm{dopamine}+\mathrm{dobutamine}\right)\times 1+\mathrm{milrinone}\times 30+\left(\mathrm{epinephrine}+\mathrm{norepinephrine}\right)\times 100$.

The transthoracic echocardiographic follow-up was done at one week, one month and six months after surgery by an experienced cardiology team. Postoperative echocardiographic parameters primarily stressed on the status of reconstructed pulmonary valve, RVOT gradient and degree of PR. Echocardiographic parameters for PR were based on recommendations of the European Association of Echocardiography and included PR jet width compared to pulmonary annulus, color flow PR jet width, jet density and deceleration rate-continuous wave, pulmonary systolic flow compared to systemic flow-pulse wave, jet width/RV outflow diameter, diastolic flow reversal, regurgitation fraction, PR index, pulmonary valve and RV size. Echocardiographc study was performed in all patients.

The data were analyzed using SPSS version 21 (SPSS, Inc., Chicago, IL, USA). Data were presented as mean (standard deviation), unless stated otherwise. Normality of the data was tested by one-sample Kolmogorov-Smirnov test and parametric or nonparametric statistics were used accordingly. Comparison of between-group binomial data was done using Fisher’s exact test. Intragroup comparison was performed using paired samples Student’s t-test or Wilcoxon test, and intergroup comparisons were made using Mann-Whitney test or independent samples Student’s t-test based on normality. Bonferroni correction was made for multiple comparisons. All P-values <0.05 were considered statistically significant.

## RESULTS

### Preoperative Baseline Characteristics

Gender distribution in the PTFEV group was nine males and six females, whereas 12 males and 3 females were in the TAP group with male preponderance in the study population. The mean age in the PTFEV group was 7.9 years compared to 6.06 years in the TAP group. The mean weight in the PTFEV and TAP group was 18.27 kg and 16.38 kg, respectively. Body surface area was comparable in both groups (0.76 kg/m2 in the PTFEV group and 0.68 kg/m2 in the TAP group). Room air saturation was 79% in the PTFEV group and 80% in the TAP group. Hemoglobin levels were 17.4 g/dl in the PTFEV group and 15 g/dl in the TAP group. One patient in the TAP group had a palliative Blalock-Taussig (BT) shunt before the definitive correction (Table 1). All patients had sinus rhythm with right ventricular hypertrophy in the electrocardiogram. Right ventricular outflow tract and pulmonary artery dimensions were comparable in both groups, except for two patients in the PTFEV group who had significant post-stenotic dilatation of right pulmonary artery (P<0.05) (Tables 2 and 3).

**Table 1:** Preoperative baseline characteristics.

Parameter		Group	n	Mean±SD	*P*-value
Gender	Male	PTFEV	9		
		TAP	12		
	Female	PTFEV	6		
		TAP	3		
Age (years)		PTFEV	15	7.91±6.52	0.39 (Mann-Whitney test)
		TAP	15	6.06±5.50	
Weight (kg)		PTFEV	15	18.27±10.58	0.20 (Mann-Whitney test)
		TAP	15	16.38±10.06	
BSA (kg/m^2^)		PTFEV	15	0.76±0.29	0.20 (Mann-Whitney test)
		TAP	15	0.68±0.30	
spO_2_		PTFEV	15	79.68±11.13	0.73 (Student's t-test)
		TAP	15	80.80±5.34	
Hemoglobin (gm/dl)		PTFEV	15	17.45±4.85	0.11 (Student's t-test)
		TAP	15	15.04±2.80	
Hematocrit (%)		PTFEV	15	53.04±16.28	0.19 (Student's t-test)
		TAP	15	46.04±9.77	
Preoperative BT shunt		PTFEV	0		
		TAP	1		

BSA=body surface area; BT=Blalock-Taussig; PTFEV=polytetrafluoroethylene valve; spO_2_=oxygen saturation; TAP=transannular patch

**Table 2:** Preoperative pulmonary artery dimensions based on transthoracic echocardiography.

Structure	Group	n	Minimum (mm)	Maximum (mm)	Mean (mm)	*P*-value
Pulmonary annulus	PTFEV	15	5	12	7.820	0.08
	TAP	15	7	14	9.133	
MPA	PTFEV	15	7	40	12.393	0.95
	TAP	15	7	16	9.933	
RPA	PTFEV	15	3.5	40	10.707	0.045
	TAP	15	4	11	6.953	
LPA	PTFEV	15	4.3	35	9.727	0.41
	TAP	15	4	14	7.553	

LPA=left pulmonary artery; MPA=main pulmonary artery; PTFEV=polytetrafluoroethylene valve; RPA=right pulmonary artery; TAP=transannular patch

**Table 3:** Preoperative pulmonary artery dimensions based on cardiac catheterization study.

Structure	Group	n	Minimum (mm)	Maximum (mm)	Mean (mm)	*P*-value
Pulmonary annulus	PTFEV	15	5	12	7.6	0.301
	TAP	15	7	12	8.5	
MPA	PTFEV	15	4.5	38	12.4	0.73
	TAP	15	8	15	10.3	
RPA	PTFEV	15	7	38	12	0.01
	TAP	15	5	11	7.3	
LPA	PTFEV	15	3.8	37	11.2	0.09
	TAP	15	5	16.5	7.7	

LPA=left pulmonary artery; MPA=main pulmonary artery; PTFEV=polytetrafluoroethylene valve; RPA=right pulmonary artery; TAP=transannular patch

### Intraoperative and Postoperative Immediate Outcomes

Gradient across the RVOT was greater in the PTFEV group (18.07±10.82 mmHg vs. 12.20±11.80 mmHg), but it was not statistically significant. Severe PR was present in all cases in the TAP group compared to six cases having mild PR in the PTFEV group and nine cases with none. Tricuspid annular plane systolic excursion (TAPSE) was comparable in both groups (PTFEV 16.20±2.42 vs. TAP 16.80±2.01, P=0.7). Peak RV/LV ratios were similar in both groups (PTFEV 0.55±0.10 vs. TAP 0.55±0.08, P=0.87) (Table 4). MAPCAs were present in 16 patients, nine in the TAP group and seven in the PTFEV group. Intraoperative MAPCA clipping did not affect the duration of surgery, bypass time and cross-clamp time in both groups.

**Table 4:** Intraoperative and postoperative immediate outcomes.

		PTFEV (n=15)	TAP (n=15)	*P*-value
Duration of surgery (min)		324±89.28	313±79.47	0.93
CPB time (min)		190±50.25	190±63.48	0.88
Cross-clamp time (min)		146±46.37	147.47±54.20	0.93
RVOT gradient (mmHg)		18.07±10.82	12.20±11.80	0.06
Pulmonary regurgitation	Absent	9	0	
	Mild	6	0	
	Moderate	0	0	
	Severe	0	15	
TAPSE (cm)		16.20±2.42	19±2.01	0.74
Peak RV/LV ratio		0.55±0.10	0.55±0.08	0.87
Vasoactive inotropic score		11.03±9.8	12.80±9.89	0.47
Extubation time (hours)		12.93±7.55	22.23±15.11	0.04
ICU stay (days)		4.67±1.95	5.93±3.01	0.17

CPB=cardiopulmonary bypass; ICU=intensive care unit; LV=left ventricle; PTFEV=polytetrafluoroethylene valve; RV=right ventricle; RVOT=right ventricular outflow tract; TAP=transannular patch; TAPSE=tricuspid planar systolic excursion

The PTFEV group had significantly lower central venous pressure (CVP) in the immediate postoperative period compared to the TAP group (7.60±2.06 vs. 10.13±1.73, P=0.002). The need for inotropes as measured by the vasoactive inotropic score (VIS) was lower in the PTFEV group compared to the TAP group (11.03±9.8 vs. 13.80±9.89), but it was not statistically significant (P=0.47). Extubation time was significantly shorter in the PTFEV group compared to the TAP group (12.93±7.55 hrs vs. 22.23±15.11 hrs, P=0.04) (Table 4). The ICU length of stay of patients undergoing PTFEV replacement was shorter (4.67±1.95 days) compared to the TAP group (5.93±3.01 days), but it was not statistically significant (P=0.17) (Table 4). Two patients in the TAP arm were reintubated, one due to pulmonary hemorrhage and the other due to carbon dioxide retention. No patient had clinically significant residual VSD in both groups. There was no in-hospital mortality.

### Six-Month Echocardiographic Follow-Up

The PTFEV group maintained a higher gradient across the RVOT in comparison to the TAP group throughout the study duration, although it was not statistically significant. During the six-month period, both groups showed a decreasing trend in the RVOT gradient, although the difference was not statistically significant. At the start of the follow-up, the PTFEV group had higher gradients when compared to the TAP group (18.07±10.82 mmHg vs. 12.20±11.80 mmHg, P=0.06). However, the gradients at the end of the study were comparable (PTFEV=8.40±8.95 mmHg vs. TAP=8.33±15.52 mmHg, P=0.6), although not statistically significant (Table 5). PR in the PTFEV group was absent in five patients at 24 hours after surgery, and this increased to six patients over the six-month follow-up period. Out of three patients with trivial PR in PTFEV group at 24 hours after surgery, one patient progressed to mild regurgitation during the six-month follow-up period. At the study endpoint, PR was absent in six, trivial in one and mild in eight patients in the PTFEV group. In the TAP group, severe PR was persistent throughout the follow-up period in all patients (n=15) (Table 6). There was no statistically significant correlation of PR amongst the two groups. There was no mortality at six months of follow-up.

**Table 5:** Progression of the right ventricular outflow tract gradient.

RVOT gradient	Group	Mean SD	*P*-value (Mann-Whitney test)
Intraoperative	PTFEV	18.07±10.82	0.06
	TAP	12.20±11.80	
24 hours	PTFEV	14.80±13.10	0.139
	TAP	9.13±11.12	
7 days	PTFEV	13.13±10.55	0.116
	TAP	9.40±12.57	
1 month	PTFEV	10.33±9.83	0.135
	TAP	8.53±13.82	
6 months	PTFEV	8.40±8.95	0.597
	TAP	8.33±15.52	

PTFEV=polytetrafluoroethylene valve; TAP=transannular patch

**Table 6:** Progression of pulmonary regurgitation.

Pulmonary regurgitation	24 hrs		7 days		1 month		6 months	
	PTFEV	TAP	PTFEV	TAP	PTFEV	TAP	PTFEV	TAP
Absent	5	0	6	0	6	0	6	0
Trivial	3	0	2	0	1	0	1	0
Mild	7	0	7	0	8	0	8	0
Severe	0	15	0	15	0	15	0	15

PTFEV=polytetrafluoroethylene valve; TAP=transannular patch.

## DISCUSSION

Transannular patch augmentation of RVOT in surgical correction of TOF is invariably associated with PR and causes RV dysfunction in long-term follow-up. Different designs of PTFE pulmonary valve substitutes to address PR have been constructed and their performance studied. Nunn et al.^[3]^ devised a PTFE bicuspid valve design with the leaflet commissures aligned at an oblique long axis of the RVOT. Further refinements in design with the construction of tricuspid PTFE valves within conduits have been described by Ando et al.^[5]^, as well as the construction of sinuses in the PTFE tricuspid pulmonary graft conduit by Miyazaki et al.^[6]^.

The construction of PTFE bicuspid valves does not increase cardiopulmonary bypass or total operative time in our study. Our patients with PTFE valves had significantly lower CVP levels in the immediate postoperative period (7.60 vs. 10.13). Patients in the PTFEV group had earlier extubation time (12.9 hrs vs. 22.2 hrs, P=0.04) in our study, which was statistically significant. These findings were similar to the study by Sasson et al.^[7]^ (monocusp valves - 24 hrs vs. TAP - 36 hrs) and Sasikumar et al.^[2]^ (monocusp group - 8.7 hrs vs. TAP - 11 hrs) (Table 7).

**Table 7:** Other studies versus the present study comparing postoperative ventilation time/ICU stay.

Study	Group	Ventilation time (hours)	ICU stay (days)
Sasson et al.^[7]^	PTFE membrane monocusp	24	4
	Transannular	36	8
Sasikumar et al.^[2]^	PTFE membrane monocusp	8.7	3
	Transannular	11.0	4
Present study	PTFE membrane valve with Nunn's modification	12.9	4.67
	Transannular	22.2	5.93

ICU=intensive care unit; PTFE=polytetrafluoroethylene

The fall in RVOT gradient in the PTFEV group was greater and approached the TAP group within six months of follow-up (PTFEV 18.07 to 8.40 vs. TAP 12.20 to 8.33) in our study. Gil-Jaurena et al.^[8]^ reported similar gradients in the outflow tract (21.80 mmHg) at discharge. The RVOT gradients were comparable as reported by Quintessenza et al.^[9]^ (maximum gradient of 20 mmHg) at six months of follow-up.

Quintessenza et al.^[9]^, in their study of 42 out of 126 patients who received the 0.1 mm PTFE bicuspid valve and were followed up for three years reported 28 patients (66%) with grade 3 (moderate) PR. Nunn et al.^[3]^, in their follow-up of 25 patients over 2.7 years, showed 93% of the cases having mild pulmonary incompetence. Gil-Jaurena et al.^[8]^, in their follow-up of 21 patients with bicuspid PTFE valves, reported 19 (90%) patients with mild and 2 (10%) patients with moderate pulmonary incompetence at discharge. Sasson et al.^[7]^, in their follow-up of 30 patients with monocusp implantation over seven months to five years reported mild PR in 62.9%, moderate PR in 22.3% and severe PR in 14.8% cases (Table 8). Brown et al.^[10]^ showed midterm results obtained one year after PTFE monocusp valve insertion; the trace-to-mild degree of PI was detected in 60% of all patients, with moderate-to severe PR in only 13%. In our study, PR was absent in six (40%), trivial in one (7%) and mild in eight (53%) patients at six months of follow-up. None of the patients had moderate or severe pulmonary incompetence over the six-month follow-up.

**Table 8:** Other studies versus the present study comparing severity of pulmonary regurgitation at the end of the follow-up period.

Study	Follow-up period	Type of valve reconstructed	Pulmonary regurgitation (in % of cases)			
			Absent or trivial	Mild	Moderate	Severe
Sasson et al.^[7]^	7 months to 5 years	PTFE membrane monocusp	0	62.9	22.3	14.8
Brown et al.^[10]^	1 year	PTFE membrane monocusp	0	60	27	13
Sasikumar et al.^[2]^	1 year	PTFE membrane monocusp	81.25	12.5	6.25	0
Nunn^[3]^	0.7-2.7 years	PTFE membrane valve with Nunn's modification	16.7	75	8.3	0
Present study	6 months	PTFE membrane valve with Nunn's modification	60	40	0	0

PTFE=polytetrafluoroethylene

## CONCLUSION

In our experience, bicuspid valve using 0.1 mm PTFE membrane is inexpensive, feasible, easy to construct and does not prolong the operative time significantly. These valves achieve a high degree of pulmonary competence and does not increase the RVOT gradient over the short-term follow-up. They produce immediate relief from PR, significantly decrease the CVP in immediate postoperative period, facilitate early extubation, and thus prevent ventilator-related comorbidities. The effect of bicuspid PTFE membrane valves on RV function needs a long-term follow-up. However, this was a single-center study, which limits its generalizability, and also included a relatively small number of patients with short-term follow-up. A long-term follow-up study is required to confirm these findings and to compare them with the results obtained using other techniques.

**Table:** 

Authors' roles & responsibilities	
SR	Substantial contributions to the conception or design of the work; or the acquisition, analysis or interpretation of data for the work; agreement to be accountable for all aspects of the work in ensuring that issues related to the accuracy or integrity of any part of the work are appropriately investigated and resolved; final approval of the version to be published
VJ	Substantial contributions to the conception or design of the work; or the acquisition, analysis or interpretation of data for the work; drafting the work or revising it critically for important intellectual content; final approval of the version to be published
SKST	Substantial contributions to the conception or design of the work; or the acquisition, analysis or interpretation of data for the work; drafting the work or revising it critically for important intellectual content; final approval of the version to be published
HS	Substantial contributions to the conception or design of the work; or the acquisition, analysis or interpretation of data for the work; drafting the work or revising it critically for important intellectual content; final approval of the version to be published
SM	Substantial contributions to the conception or design of the work; or the acquisition, analysis or interpretation of data for the work; drafting the work or revising it critically for important intellectual content; final approval of the version to be published
RLK	Substantial contributions to the conception or design of the work; or the acquisition, analysis or interpretation of data for the work; final approval of the version to be published
GDP	Substantial contributions to the conception or design of the work; or the acquisition, analysis or interpretation of data for the work; drafting the work or revising it critically for important intellectual content; design and critical revision; final approval of the version to be published
MKR	Substantial contributions to the conception or design of the work; or the acquisition, analysis or interpretation of data for the work; drafting the work or revising it critically for important intellectual content; final approval of the version to be published

## References

[1] Greutmann M, Tobler D (2012). Changing epidemiology and mortality in adult congenital heart disease: looking into the future. Future Cardiol.

[2] Sasikumar D, Sasidharan B, Tharakan JA, Dharan BS, Mathew T, Karunakaran J (2014). Early and 1-year outcome and predictors of adverse outcome following monocusp pulmonary valve reconstruction for patients with tetralogy of Fallot: a prospective observational study. Ann Pediatr Cardiol.

[3] Nunn GR, Bennetts J, Onikul E (2008). Durability of hand-sewn valves in the right ventricular outlet. J Thorac Cardiovasc Surg.

[4] Wernovsky G, Wypij D, Jonas RA, Mayer JE Jr, Hanley FL, Hickey PR (1995). Postoperative course and hemodynamic profile after the arterial switch operation in neonates and infants. A comparison of low-flow cardiopulmonary bypass and circulatory arrest. Circulation.

[5] Ando M, Takahashi Y (2009). Ten-year experience with handmade trileaflet polytetrafluoroethylene valved conduit used for pulmonary reconstruction. J Thorac Cardiovasc Surg.

[6] Miyazaki T, Yamagishi M, Maeda Y, Yamamoto Y, Taniguchi S, Sasaki Y (2011). Expanded polytetrafluoroethylene conduits and patches with bulging sinuses and fan-shaped valves in right ventricular outflow tract reconstruction: multicenter study in Japan. J Thorac Cardiovasc Surg.

[7] Sasson L, Houri S, Raucher Sternfeld A, Cohen I, Lenczner O, Bove EL (2013). Right ventricular outflow tract strategies for repair of tetralogy of Fallot: effect of monocusp valve reconstruction. Eur J Cardiothorac Surg.

[8] Gil-Jaurena JM, Ferreiros M, Castillo R, Conejo L, Cuenca V, Zabala JI (2010). Use of a pulmonary neovalve with a transannular patch for repair of tetralogy of fallot. Rev Esp Cardiol.

[9] Quintessenza JA, Jacobs JP, Chai PJ, Morell VO, Lindberg H (2010). Polytetrafluoroethylene bicuspid pulmonary valve implantation: experience with 126 patients. World J Pediatr Congenit Heart Surg.

[10] Brown JW, Ruzmetov M, Vijay P, Rodefeld MD, Turrentine MW (2007). Right ventricular outflow tract reconstruction with a polytetrafluoroethylene monocusp valve: a twelve-year experience. J Thorac Cardiovasc Surg.

